# Commentationes Societatis Regiæ Scientiarum Gottingensis

**Published:** 1781-08

**Authors:** 


### THE LONDON MEDICAL JOURNAL,

### For AUGUST 1781.

### SECTION I.

### BOOKS.

I. *Commentationes Societatis Regiæ Scientiarum Gottingensis.* Classis Physicæ, Tomus I. ad A. 1778. 4to. Gottingæ, 1779. 139 pages, with six copper plates.

THE works published every year by learned Societies in different parts of Europe are so numerous, and the subjects discussed in them so various, that the printing each class of papers separately in the manner adopted by the Royal Society at Gottingen, would be extremely agreeable and convenient to men of science. In the class of *Physica* the society include medical as well as phiphilosophical papers. The articles of the present collection are as follows:

I. *An account of a præternatural and uncommon adhesion of the rectum to the urinary bladder, and of a consequent defect of anus. By* Henry Augustus Wrisberg, M. D. *professor of anatomy.*

II. *Anatomical remarks on the descent of the testicles from the abdomen into the scrotum, with a view to illustrate the doctrine of the hernia congenita in either sex.* By the same.

These two papers having been published separately by the author have been already noticed in our Journal. [See vol. i. p. 375, & seq.]

III. *Remarks on several newly discovered plants. By* John Andrew Murray, M. D. *professor of botany in the university of Gottingen.*

The author begins with deploring the irreparable loss botany has sustained by the deaths of Bernard de Jussieu, Haller, and Linnæus, on each of whose memories he bestows a grateful eulogium. Speaking of the latter, he observes, that the world was deprived of him at a time when our knowledge of plants was beginning to be wonderfully enlarged by the travels of ingenious botanists, as Aublet, Sonnerat, Pallas, Solander, Forster, Sparrman, and Thunberg, into into the remoteſt parts of the globe. He laments, however, that ſince the death of Linnæus the treaſures collected by thoſe gentlemen have remained '*projecta, diſperſa, et quaſi derelicta*;' and he expreſſes his fears, that in deſcribing new plants a confuſion will be introduced into the ſcience of botany, which may laſt for many years, till a ſecond Linnæus ariſes to diſpel it. In order to prevent this evil from taking place, he intreats the lovers of botany to avail themſelves of every opportunity of procuring accurate deſcriptions of ſuch plants as are new, or hitherto not generally known. With this view the author has ſelected ſeveral plants cultivated in the botanic garden at Gottingen, none of which, to his knowledge, have as yet been perfectly deſcribed. The firſt three are ſpecies of *Salvia.* Dr. Murray obſerves, that many ſpecies of this genus reſemble each other ſo nearly, that it is no eaſy taſk to aſcertain the characteriſtic difference of each. Zinn, who directed his attention to this genus with his uſual induſtry, deſcribed twenty-ſix ſpecies; but ſince his time the number has ſo much increaſed, that in the laſt edition of Linnæus's *Syſtema Vegetabilium* 39 ſpecies are enumerated, and ten more might now be added. Thoſe deſcribed by by our author are as follows: 1. Salvia Coccinea, *foliis cordato-oblongis obtuse serratis, calycibus tripartitis, labio corollæ inferiori amplissimo.* The seeds of this plant, which is a native of Æthiopia, were sent to him by Mr. Aiton of Kew, and it flowered in August. It differs from every other species of *Salvia* in the scarlet colour of its flowers, on which account M. de Jussieu named it *Coccinea.* 2. Salvia Nilotica, *foliis sinuatis angulatis crenato-dentatis, calycum dentibus spinosis, angulis et margine faucis ciliatis.* This plant, which was so named by M. de Jussieu, the botanist who first cultivated it, bears a small blue flower. 3. Salvia Nubia, *foliis lanceolato-ovatis duplicato-crenatis, tubo corollæ incurvato.* Dr. Murray procured the seeds of this plant from different friends, who all agreed that in the botanic garden at Paris it went by the name of *Nubia*, except one, who spoke of it as an Abyssinian plant. The other plants described by our author are, 5. Sideritis Elegans, *herbacea ebracteata villosa, caule diffuso, calycum laciniis subæqualibus, spinulosis.* Of this plant Dr. Murray observes, that it was sent to him as a species of marrubium, but proved to be a true sideritis, to which, on account of the beautiful colour of its flowers, he has given the name of *Elegans*. He can find no traces of it in any botanic writer, except Fabricius, and even this author confounds it with the *Sideritis Romana* of Linnæus. 6. PLANTAGO EXIGUA, *caule ramoso herbaceo, foliis subulatis integerrimis, capitulis foliosis.* This plant, which is a native of the East Indies, appears at first view to be a variety of the *Plantago Indica* Linn. from which however our author finds it to differ very materially in the form of its leaves. 7. SOPHORA ALBA Linn. Dr. Murray observes, that the descriptions hitherto given of this plant by Linnæus and others are defective; and that the engraving of it given by Martyn in his *Stirp. rarior.* tab. 44. and which seems to have been made from a decayed specimen, has so far imposed on Linnæus as to lead him into a mistake in his *Mant. Plant.* 2. p. 440.—For our author's accurate descriptions (illustrated by engravings) of this and the other plants already mentioned, we must refer our readers to the work itself.

IV. *Remarks on the Fistula Lachrymalis. By* Augustus Gottlieb Richter, M. D. *professor of surgery in the university of Gottingen.*

Almost all the modern writers on this disease agree in ascribing it to an obstruction of the nasal duct; but the author of these remarks contends that this cause, though it may, does in fact but rarely exist. He observes, for instance, that a fracture or caries of the bones surrounding the lachrymal sac, or an ulceration of the sac itself, which are perhaps the only causes that can lay the foundation of such an obstruction, will seldom be found to precede the disease in question; and that inspissated mucus, acrimony of the tears, and other similar causes which have been supposed to occasion it, are totally inadequate to such an effect. Upon this principle he reprobates the use of all the instruments which have been invented of late years in different countries, particularly in France, for the purpose of removing the supposed obstruction. They are most of them, he thinks, calculated rather for shew than real utility; and what is more, there are but very few, even of the most dexterous surgeons, who are able to use them.

" Every candid practitioner, says our author, " who has seen this disease, must confess, and " I myself, who have frequently had occasion " to see it, acknowledge, that we are seldom " able to cure it perfectly. It frequently re- " turns after the *ductus ad nares* has been care-

" fully and completely opened; and many pa-

" tients are as much troubled with a defluxion of

" tears after, as they were before the operation;

" which very often affords them but little or

" no sensible relief. I have even seen patients

" in whom after the most dexterous operation

" the lachrymal sac not only continued tumid,

" is adopted, the disease generally returns, and

" returns, in my opinion, because the cause of

" it is not removeable by this operation. I

" have repeatedly continued the use of the

" bougie eight or ten weeks, and in two cases

" half a year, notwithstanding which the dis-

" ease constantly returned as soon as the wound

" in the sac was healed. In two patients I was

" induced, by a return of the symptoms, to

" repeat the operation a second time, and in

" both

" both instances I was convinced there was no

" obstruction, as a probe passed readily into the

After having thus combated these commonly received notions concerning the nature and cure of this disease, Dr. Richter proceeds to deliver his own ideas on the subject. He begins by distinguishing three species of fistula lachrymalis, the cause and cure of each of which is materially different. The first and least frequent of these is is occaſioned by an obſtruction of the lachrymal ſac; the ſecond, and moſt common of the three, is owing to a tranſlation of morbific matter to the channel through which the tears are to paſs; the third and laſt ariſes from a weakneſs of the lachrymal ſac. He treats pretty fully of the ſymptoms and treatment of each of theſe ſpecies.

He obſerves, that if the diſeaſe remains uniformly the ſame; if, by compreſſion, no fluid is forced into the noſtrils; if, in the early ſtage of it, the lachrymal ſac is neither painful nor inflamed, and the fluid that regurgitates from it is a colourleſs mucus or water, we may reaſonably aſcribe the complaint to an obſtruction of the duct, provided theſe ſymptoms have been preceded by any cauſe likely to produce ſuch an effect. But he thinks it will ſeldom happen that the diſeaſe, ariſing from ſuch a cauſe, will paſs on to what he terms the ſecond ſtage, in which the ſac inflames, and the fluid contained in it aſſumes a puriform appearance. He allows, however, that this may ſometimes happen through miſmanagement or any accidental cauſe exciting inflammation.

In this ſpecies of the diſeaſe our author recommends an inciſion to be made into the lachrymal

 ſac,

ſac, which is then to be lightly filled with lint and covered with a plaſter. Four or five days after this operation, when there is no danger of hæmorrhage, a ſmall, flexible, ſilver probe, ſimilar to that invented by M. Mejean, is to be introduced at the wound and puſhed through the duct into the noſtrils. This part of the operation, we are told, generally requires a conſiderable degree of force, and is attended with acute pain and the diſcharge of a drop or two of blood from the noſtrils. After withdrawing the probe a piece of ſmall catgut is to be paſſed into the duct, and ſuffered to remain there four or five days, at the end of which time it is to be removed and one of a greater thickneſs introduced in its ſtead. In this manner we are directed to proceed for about a month, increaſing the thickneſs of the catgut every five or ſix days, till it is equal in bulk to the natural ſize of the duct. The catgut is to be removed every morning, and the ſac cleanſed by injecting into it barley-water and honey, or any other emollient liquor. As that part of the catgut which remains in the noſtril is apt to become hard, dry, and incruſted with mucus, the drawing it upwards through the wound would be painful to the patient; we are therefore adviſed to pull it downwards through the noſtril; and, and, that this may be eaſily done, we are to take care when we introduce it that a ſufficient portion of it projects from the duct within the noſe.

At the end of about four weeks the emollient is to be exchanged for a drying injection of lime water or Goulard's vegeto-mineral water, and inſtead of the catgut a leaden probe is to be introduced into the duct, and the uſe of it continued for two months, or till there is no longer any appearance of pus, and till a fluid injected into the ſac paſſes readily and copiouſly into the noſtrils, or the patient's breath, when his mouth and noſtrils are ſhut, forces its way with violence through the wound in the ſac.

Dr. Richter obſerves, that ſeveral practitioners, particularly Mr. Warner, prefer the making an artificial vent for the tears, by perforating the os unguis, upon a ſuppoſition that by this means a return of the complaint is leſs likely to happen than by attempting to remove the obſtruction of the duct. Our author, however, is clearly of opinion, that they are miſtaken in this point, not only becauſe the diſorder does not return after the operation juſt now deſcribed, provided it was really occaſioned by obſtruction, but likewiſe becauſe this perforation of the bone, inſtead of relieving the patient, is productive of conſiderable inflamation, which ſometimes cloſes the lachrymal ducts, and totally prevents the paſſage of the tears into the ſac, which is the reaſon why a weeping eye is ſo often the effect of ſuch an operation.

The ſecond ſpecies of fiſtula lachrymalis, or that which our author aſcribes to metaſtaſis, he ſuppoſes to be owing to an acrid and morbid humour affecting chiefly the glands of the ſac.

Thus the matter of the ſmall pox, ſcrophula, gout, lues venerea, tinea capitis, &c. will frequently excite a diſeaſe of this kind. Our author ſpeaks of that which ſucceeds the ſmall pox as being the moſt difficult of cure of any. He has ſometimes ſucceeded in the ſcrophulous fiſtula lachrymalis by means of iſſues, bark, calomel, and extract of hemlock. He obſerves that the arthritic fiſtula is apt to return, particularly in ſpring or autumn; but he has in ſeveral caſes removed it by iſſues, and the uſe of aconitum, antimony, and the bark. When the diſeaſe is owing to a venereal taint it is to be cured by mercury. In two children he has ſeen it come on after the drying up of a tinea capitis, and diſappear upon the return of the original complaint.

Dr. Richter finds it difficult to account ſatiſfactorily for the manner in which a fiſtula lachrymalis originates from theſe cauſes, but having obſerved that the lachrymal duct is extremely irritable, ſo as to be capable of conſiderable contraction, he thinks it likely that in caſes of this ſort the diſeaſe is owing to ſpaſm; and that upon this principle we may eaſily explain why it ſeldom remains long in the ſame ſtate, but is ſometimes better and at other times worſe.

The third ſpecies of fiſtula lachrymalis, according to our author, is owing to a ſtate of atony, which prevents the lachrymal ſac from propelling the tears into the noſtrils. He obſerves, however, that this is ſeldom a primary complaint, being generally produced by one or other of the two ſpecies already ſpoken of. The only remedies recommended in caſes of this ſort are ſtrengthening applications, and gentle preſſure, to prevent too great a diſtenſion of the ſac.

V. *A Diſcourſe on the Hiſtory of Alum. By* John Beckmann.

We have here a very elaborate hiſtory of a ſalt with which, in the opinion of the learned author, the antients were unacquainted, as the ſubſtances to which they gave the names of συπτηρίας and *alumen*, were only preparations of vitriol. The ſalt at preſent diſtinguiſhed by the name name of *alum* was for several centuries imported into Europe from the East, where it was first invented. It is difficult to ascertain the precise date of the discovery, but our author supposes it to have been made in the 12th century, about which time likewise the term *vitriol* first came into use; Albertus Magnus being the first writer who adopted it.

The author takes occasion to notice the diversity of opinions that has prevailed concerning the etymology of *Roch alum*; and, after examining each of them, adopts that of Leibnitz, who supposes, that the word *Rocca* is derived from a place of that name in Syria, from whence the art of preparing alum is said to have been first brought into Italy about the year 1458.

